# Model Based Evaluation of Hypersensitivity Adverse Drug Reactions to Antimicrobial Agents in Children

**DOI:** 10.3389/fphar.2021.638881

**Published:** 2021-04-30

**Authors:** Abdelbaset A. Elzagallaai, Michael J. Rieder

**Affiliations:** ^1^ Department of Paediatrics, London, ON, Canada; ^2^ Physiology and Pharmacology, Schulich School of Medicine and Dentistry, Western University, London, ON, Canada

**Keywords:** children, adverse drug reactions, modeling, antimicrobacterial, drug safety

## Abstract

Drug use in children is–in most cases–supported by extrapolation of data generated from clinical trials in adult populations. This puts children at higher risk of developing adverse drug reactions (ADRs) due to “off-label” use of drugs and dosing issues. Major types of ADRs are drug hypersensitivity reactions, an idiosyncratic type of ADRs that are largely unpredictable and can cause high morbidity and mortality in a hard-to-identify specific population of patients. Lack of a complete understanding of the pathophysiology of DHRs and their unpredictive nature make them problematic in clinical practice and in drug development. In addition, ethical and legal obstacles hinder conducting large clinical trials in children, which in turn make children a “therapeutic orphan” where clear clinical guidelines are lacking, and practice is based largely on the personal experience of the clinician, hence making modeling desirable. This brief review summarizes the current knowledge of model-based evaluation of diagnosis and management of drug hypersensitivity reactions (DHRs) to antimicrobial drugs in the pediatric population. Ethical and legal aspects of drug research in children and the effect of different stages of child development and other factors on the risk of DHRs are discussed. The role of animal models, *in vitro* models and oral provocation test in management of DHRs are examined in the context of the current understanding of the pathophysiology of DHRs. Finally, recent changes in drug development legislations have been put forward to encourage drug developers to conduct trials in children clearly indicate the urgent need for evidence to support drug safety in children and for modeling to guide these clinical trials.

## Introduction

An adverse drug reaction (ADR) is defined as any noxious and unintended response to a drug, which occurs at doses normally used in man for prophylaxis, diagnosis or therapy of disease or modification of physiological function. A conservative estimate of the rate of ADRs in the general population is 5% per course of treatment, however; it can be as high as 50%, for example, in the case of cancer chemotherapy (Elzagallaai et al., 2017). ADRs are a leading cause of morbidly and mortality in patients from all age groups (Lombardi et al., 2018; Lombardi et al., 2019; Lombardi et al., 2020; Pagani et al., 2021). Serious ADRs occur at a rate of 6.7% in hospitalized patients and 0.32% of them are fatal. A review of 17 prospective studies of incidence rate of ADRs in pediatric in- and out-patients estimated the incidence to be 9.5% (95% CI 6.81, 12.26) in in-patients and 1.5% (95% CI 0.7, 3.03) in out-patients (Impicciatore et al., 2001). Accurate estimation of the incidence of ADRs in children is hindered by under-reporting, lack of clear definition of age groups and causality issues (Smyth et al., 2012). It is well known that subtle age differences during infancy and childhood–which extends from birth to the onset of puberty–are associated with significant changes in pharmacokinetics and pharmacodynamics of medications, notably in the first several years of life. Body weight doubles by 5 months and triples by 1 year. Major maturation of body systems occur during the first few years of life and body water and fat compositions changes dramatically (Kauffman, 2019). All these factors put children at risk of developing ADRs as typically drugs are not studied in children during the process of drug development and approval and, hence safety data in this age group is almost always missing.

Drug Hypersensitivity reactions (DHRs) including true “drug allergy” represent up to one third of all ADRs and can be severe and life-threatening requiring prolonged hospitalization and associated healthcare costs (Demoly et al., 2007; Sousa-Pinto et al., 2020). DHRs are classified, according to their onset and the immune mechanism involved, into immediate-type DHR (IDHRs) or delayed-type DHRs (DDHRs). IDHRs occur within an hour of drug exposure and are mediated by IgE antibodies generated against the drug or metabolite(s). On the other hand, DDHRs occur days or weeks after drug exposure and are IgG or T-cell-mediated (Rieder, 2018). Antibiotics are the most commonly prescribed drugs in children (Stam et al., 2012; Holstiege and Garbe, 2013; Youngster et al., 2017) and they are the second leading cause of ADRs resulting in emergency department visit and/or hospital admission in children (18%) after cancer chemotherapy (Langerova et al., 2014; Lombardi et al., 2018; Lombardi et al., 2019; Lombardi et al., 2020; Pagani et al., 2021). DHRs represent a major clinical problem because of their potential seriousness and high morbidity. In addition, labeling a child with antibiotic allergy without confirmation has its consequences to both the patient and public health (Tanno et al., 2018). Approximately 10% of children are reported by their parents to have antibiotic allergy and 75% of them are diagnosed before their third birthday (Vyles et al., 2017b). Unfortunately, this is frequently incorrect and over-Labeling of antibiotic allergy has been demonstrated to have a negative health impact both on the patient and the health care system (Charneski et al., 2011). Unconfirmed childhood allergy labeling most often extends to the rest of the patients’ life leading to unnecessarily depriving them from useful and safe drugs and exposing them to less safe and more expensive alternatives. In fact, studies have shown that when labeled children are challenged with the culprit drug, over 90% are able to tolerate the drug (Rebelo Gomes et al., 2008; Vezir et al., 2014; Vyles et al., 2017a). Current data shows that up to 10% of children are reported to have beta-lactam allergy and are the most common trigger of anaphylactic reactions in children (Gomes et al., 2016; Regateiro et al., 2020). The risk of fatal anaphylaxis due to penicillin use has been estimated at 0.0015–0.002% of treated patients (International Collaborative Study of Severe Anaphylaxis, 2003). Up to 75% of fatal drug-induced anaphylaxis in the United States are caused by penicillins, which corresponds to 500–1,000 annual fatality (Neugut et al., 2001).

Prescribing medicines to children is challenging due to the lack of reliable safety data as a result of the limited number of clinical trials in the pediatric population. One example is dose. Dose calculation for pediatric patients based on weight and body surface area (BSA) can be both inaccurate and prone to errors. Children are not merely little adults; they have their own unique pharmacokinetics and pharmacodynamics and these parameters change dramatically especially during the first few years of life (Elzagallaai et al., 2021). Dose estimation from adult studies can be extrapolated with allometric scaling but this may not always result in an optimal or safe dose due to the variability imposed by ontogeny.

The term “off-label” use refers to the use of a drug for an indication that is not listed in the drug license and monograph (Frattarelli et al., 2014). Off-label use is very common in children, which has been demonstrated to be an independent risk factor for ADRs (Neubert et al., 2004). Off-label use of medications in children ranges from 11 to 80% and a higher rate of use has been described in younger children and in hospital setting as opposed to ambulatory care. This number increases to up to 90% in neonates in Neonatal Intensive Care Units (Conroy et al., 1999; Langerova et al., 2014). It still unclear why off-label use of drugs in children increases risk for ADRs and larger studies in this field and further research are needed (Mason et al., 2012). Hence children have been historically considered to be “therapeutic orphans” because of the lack of robust data on the safety and efficacy of drug use in children (Shirkey, 1999; Shirkey, 2006).

Historically, children have been the preferred subjects to conduct drug development research–especially vaccine research–as they were considered easy to control and previously unexposed to immune-modulating infections. For example, Edward Jenner first tested the smallpox vaccine on his own children. Jenner’s vaccine was later tested in Philadelphia in 1802 on 48 children (Burns, 2003). However, this changed dramatically after events such as Elixir of Sulfanilamide Tragedy and the Thalidomide Disaster (Shirkey, 1999). Changes in drug regulation resulted in the unintended but very real consequence of children being excluded from most drug research, resulting in the ‘therapeutic orphan’ status described above (Wilson, 1999). Over the past 2 decades this has been increasingly recognized as a problem and legislative and policy changes have addressed this. Currently, drug approval regulations and ethical principles have facilitated the enrollment of children in many trials of new drugs (NICHD, 2020). However as noted above this does not apply to older drugs, which are the most commonly prescribed to children. This review discusses the current knowledge of model-based evaluation of safety of antimicrobials in children highlighting the available models to expand our knowledge and capability to predict and mange ADRs in children.

## Pathophysiology and Etiology of Drug Hypersensitivity Reactions

**FIGURE 1:**
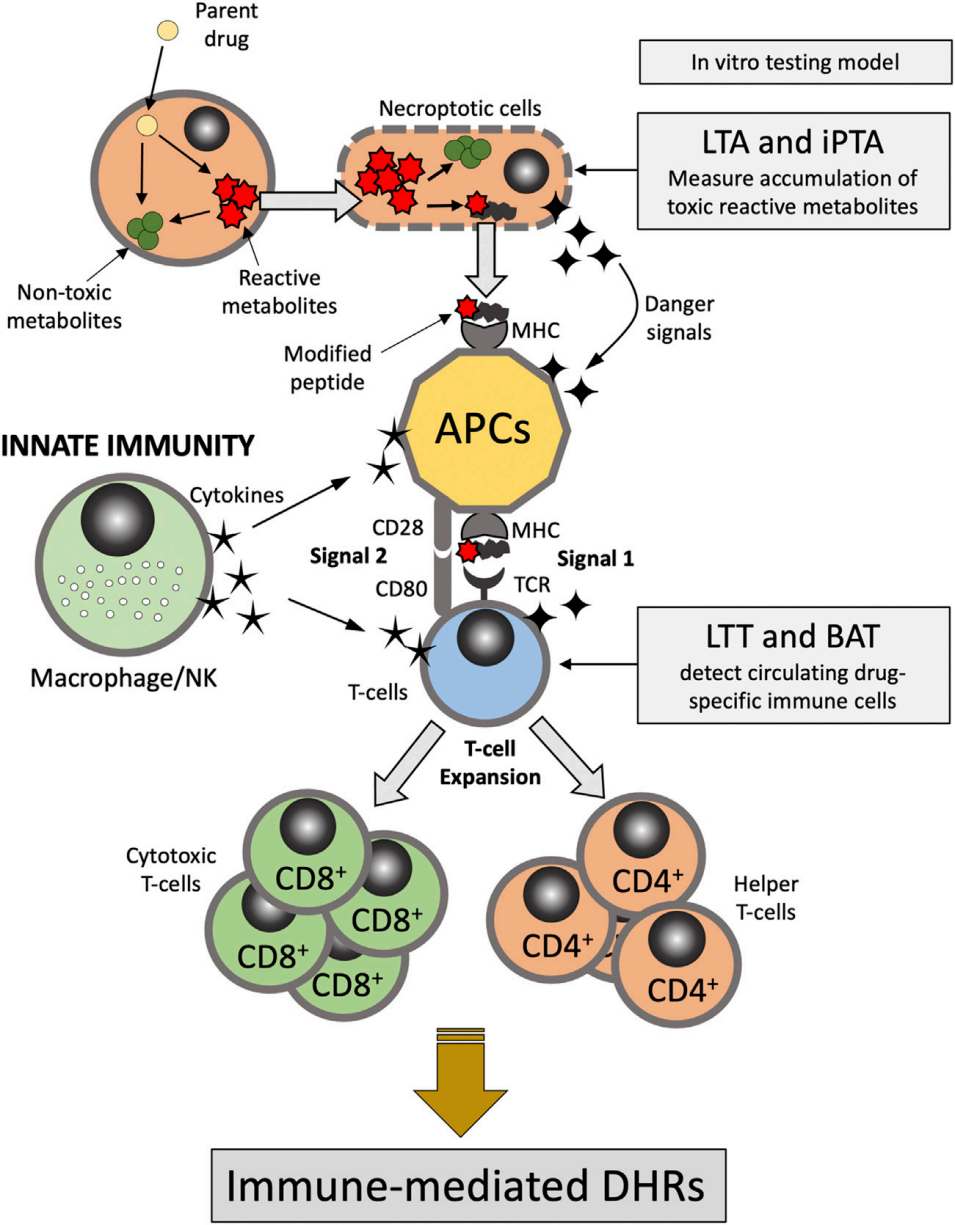
Pathophysiology of delayed-type DHRs. APC, antigen presenting cells; BAT, basophil activation test; DHRs, drug hypercreativity reactions; iPTA, *in vitro* platelet toxicity assay; LTA, lymphocyte toxicity assay; LTT, lymphocyte transformation test; MHC, Major histocompatibility complex; NK, natural killer; TCR, T-cell receptor.

There are multiple hypotheses exist that attempt at exploring the metabolic and immune mechanisms underlying DHRs. The “hapten hypothesis” proposes that drugs (or their metabolites) form covalent adducts with endogenous macromolecules (e.g., proteins), which then can be recognized by the immune system as a “non-self” antigen (Roujeau, 2006). The “danger hypothesis” assumes that in order for a full immune system response to be mounted, immune cells have to be primed by mediators released from apoptotic or necroptotic cells (dead or dying cells) (Pirmohamed et al., 2002). The “reactive metabolite hypothesis” proposes that accumulation of reactive metabolites (RMs) due to imbalance between the generation and detoxification/elimination of these RMs is the first step in the cascade of events leading to the development of the DHRs (Figure 1). In addition, “the pharmacological interaction of drugs with the immune system (p-i) hypothesis” postulates that drugs or their metabolites can directly and non-covalently interact and activate immune cells causing DHRs (Chen et al., 2018; Pichler, 2019). Evidence also exist that supports the concept of drug-induced alteration in the self-peptide repertoire presented in the context of the major histocompatibility complex (MHC) molecules by antigen presenting cells to T-cells. This has provided explanation as to the role of human leukocyte antigen (HLA) genetic variation in the pathophysiology of DHRs (e.g., abacavir-induced DHRs) (Illing et al., 2012; Ostrov et al., 2012). Understanding DHRs pathophysiology is crucial to the development and interpretation of *in vitro* tests for drug hypersensitivity as discussed further below.

## Evaluation and Management of Drug Hypersensitivity Reactions to Antimicrobials

Drug hypersensitivity always represents a major challenge to clinicians as the predicament of “to discontinue or not to discontinue” the suspected drug is often a difficult decision especially if no alternative drug is available or if alternative therapy is considered inferior in respect to outcome. Current clinical practice include detailed review of medical history; however, identifying the culprit drug may be complicated by polypharmacy. Careful physical examination and investigation of signs and symptoms including type of skin rash (e.g., urticarial, maculopapular, purpuric, bullous or eczematous) may aid differentiating drug-induced reactions from other disease conditions such as viral or bacterial infections. One method to exclude drugs is to find out whether the patient has tolerated the drug in the past, although this is not absolutely true in all cases as patients may develop reaction to drugs after taking them for years, notably in the case of Type I allergic reactions (Roberts et al., 2020).

Over the recent years several international guidelines have been released summarizing recommendations and protocols for diagnosis and management of drug allergy and hypersensitivity reactions (Gomes et al., 2016; UK NCGC, 2014; Aberer et al., 2003; Brockow et al., 2015; Cardona et al., 2020; Doña et al., 2021; Doña et al., 2018; Gupta et al., 2017; Romano et al., 2020). Other algorithms and causality assessment tools have been developed to aid identifying the causative drugs such as the Naranjo scale (Naranjo et al., 1981). Algorithms to guide management of DHRs are available and always include careful medical history, skin testing, *in vitro* testing and DPT (Ariza et al., 2020). However, the clinical presentation of DHRs is always variable and complicated by polypharmacy, concomitant infection and other diseases. In the setting of infections, diagnosis of allergy is problematic. Blood tests such as measuring serum levels of the serine protease tryptase can be helpful for diagnosis of acute type-I allergic reactions, which are immediate and IgE-mediated including anaphylactic reactions (Schwartz et al., 1989). Elevation in serum tryptase is an indicator of mast cells degranulation but the test cannot differentiate between IgE-mediated and direct mast cell degranulation and may be elevated in both anaphylactic and anaphylactoid reactions (Schwartz, 2004). Serum tryptase peaks within 1–2 h of the acute reaction, so blood samples should be obtained within this time frame, although high serum tryptase levels may last for several hours and test may still of value. In addition, the test cannot identify the culprit drug (Mirakian et al., 2009). IgE specific assays such as the RAST may be useful but are very antigen specific. In addition, there is only limited number of antigens for which RAST assays are available.

There are certain classes of antimicrobial drugs that are most associated with eliciting DHRs. These include beta-lactam antibiotics, quinolone antibiotics, sulfonamides, dapsone, vancomycin, tetracyclines, aminoglycosides, clindamycin and metronidazole (Araujo and Demoly, 2008; Elzagallaai et al., 2011a; Sanchez-Borges et al., 2013; Kuyucu et al., 2014; Grinlington et al., 2020), however, theoretically, any antimicrobial drug can cause DHRs. Clinicians must be very suspicious if one of these classes of drugs appears on the patient medical history list when untoward events occur during therapy.

Many pharmacogenetic markers have been found to associate with antimicrobial-induced DHRs. Variants in genes such as NAT2 (Wolkenstein et al., 1995; Zielinska et al., 1998; Pirmohamed et al., 2000), HLA (Lonjou et al., 2008; Kongpan et al., 2015; Wong et al., 2020), and GCLC (Wang et al., 2012) have been reported to associate with sulfonamides-induced DHRs. Other genotypes and haplotypes have been found to associate with beta-lactam-induced DHRs (Yang et al., 2006; Daly et al., 2009; Lucena et al., 2011; Gueant et al., 2015; Rutkowski et al., 2017). Specific genetic variants have also been found to put patients at higher risk of developing DHRs to cephalosporins, quinolones, macrolides, and vancomycin (Kim et al., 2009; Barbaud et al., 2014). Pharmacogenetics of DHRs to drugs including antimicrobials have been recently reviewed elsewhere (Pavlos et al., 2012; Piccorossi et al., 2020; Stocco et al., 2020).

## Modeling in Drug Therapy

Many drug regulatory agencies around the world have recently issued mandates to promote drug development for children use that is evidence-based (Turner et al., 2014). However, conducting a large-scale detailed pharmacokinetics/pharmacodynamics (PK/PD) trials in children is a huge undertake even for major pharmaceutical companies and might not be feasible. In the United States, The Best Pharmaceutical for Children Act (BPCA), which became a law in 2002, has been put forward to encourage the pharmaceutical industry to perform studies to improve evidence-based pediatric drug therapy (NICHD, 2020). Model-based studies and application of simulation and pharmacometrics for pediatric therapy has gained momentum in recent years (Vinks et al., 2015). Pharmacometrics applies quantitative mathematical models of physiology, pharmacology and pathology to predict pharmacokinetics (PK) and pharmacodynamics (PD) parameters for the purpose of assessing drug efficacy and safety (Barrett et al., 2008). This recent concept has been applied in pediatrics to evaluate the influence of growth and development on drug disposition and toxicity (Anderson et al., 2006). However, data supporting model-based evaluation of antimicrobial-induced hypersensitivity reactions has been scarce and current guidelines do highlight this problem (Doña et al., 2018; Mirakian et al., 2009; Joint Task Force on Practice Parameters et al., 2010; Mirakian et al., 2015; Muraro et al., 2017).

Application of pharmacometrics methods in clinical practice give the ability to analyze pharmacokinetics profile and define the optimal doses of drugs in special populations such as children, in whom drugs are not usually studied during clinical trials (Sutherland et al., 2019). It is also possible to quantify dose-response relationships in these populations (Lala, 2012). Pharmacometrics methods are also more cost effective than clinical trials to generate information used in optimization of drug therapy (Gobburu, 2020). This is what makes pharmacometrics methods very attractive alternative to clinical trials especially in the pediatric populations. However, accuracy of model prediction is largely dependent on the quality of the original data used during modeling (Liu and Ward, 2019).

The selection of a model is driven by the pathophysiology believed to be responsible for the ADR. For instance, in the case of DHRs there are several distinctly different immune mediated pathways producing adverse events. As eluted to in previous section, immediate events such as penicillin-induced anaphylaxis is typically mediated by IgE (Gell and Coombs Type I Hypersensitivity) (Table 1). In contrast, delayed onset DHR such as Stevens-Johnson Syndrome and Serum Sickness Like Reactions appear to be mediated by specific T cell subsets. Thus, the model system employed should be tailored around the putative pathogenesis of the ADR of interest (Figure 1). The principle of the lymphocyte toxicity assay (LTA) and the *in vitro* platelet toxicity assay (*i*PTA) tests is based on the hypothesis that DHRs are developed as a result of accumulation of toxic reactive metabolites (RMs) resulting in induction of necroptosis and release of intracellular “danger signals”, and haptenation of endogenous peptides that can be recognized by the immune system (Matzinger, 2002; Pirmohamed et al., 2002). The LTT and BAT tests detect circulating drug-specific immune cells (lymphocytes and basophil, respectively), which are thought to mediate the immune reaction (Nyfeler and Pichler, 1997; Pichler and Tilch, 2004; Hausmann et al., 2009; Marraccini et al., 2018). Role of the *in vitro* testing model is discussed further below.

**TABLE 1:** Classification of immune-mediated DHRs.

Type	Mechanism	Example	Drugs most commonly involved with DHRs
I	IgE-mediated	Anaphylaxis, urticaria, bronchospasm, and rhinitis	Beta-lactam antibiotics, ACE inhibitors, NMBAs, radiocontrast media, NSAIDs, and opioids
II	IgG-mediated	Blood cell dyscrasia	Penicillins, sulfonamides, aromatic anticonvulsants, quinine, heparin, thiazides, and gold salts
III	IgG/M-mediated	SSLR, vasculitis	Cephalosporins (e.g., cefaclor), infliximab, allopurinol, and bupropion
IV	T-cell-mediated	DRESS, SJS, TEN, AGEP, ME, and FDE	Sulfonamides, nevirapine, aromatic anticonvulsants, NSAIDs, dapsone, allopurinol, abacavir, and minocycline

ACE, angiotensin converting enzyme; AGEP, acute generalized exanthematic pustulosis; DRESS, drug rash with eosinophilia and systemic symptoms; FDE, fixed drug eruption; ME, maculopapular exanthema; NMBAs, neuromuscular blocking agents; NSAIDs, non-steroidal anti-inflammatory drugs; SJS, Steven’s Johnson syndrome; SSLR, serum sickness-like reactions; TEN, toxic epidermal necrolysis.

## Drug Provocation Test

**FIGURE 2:**
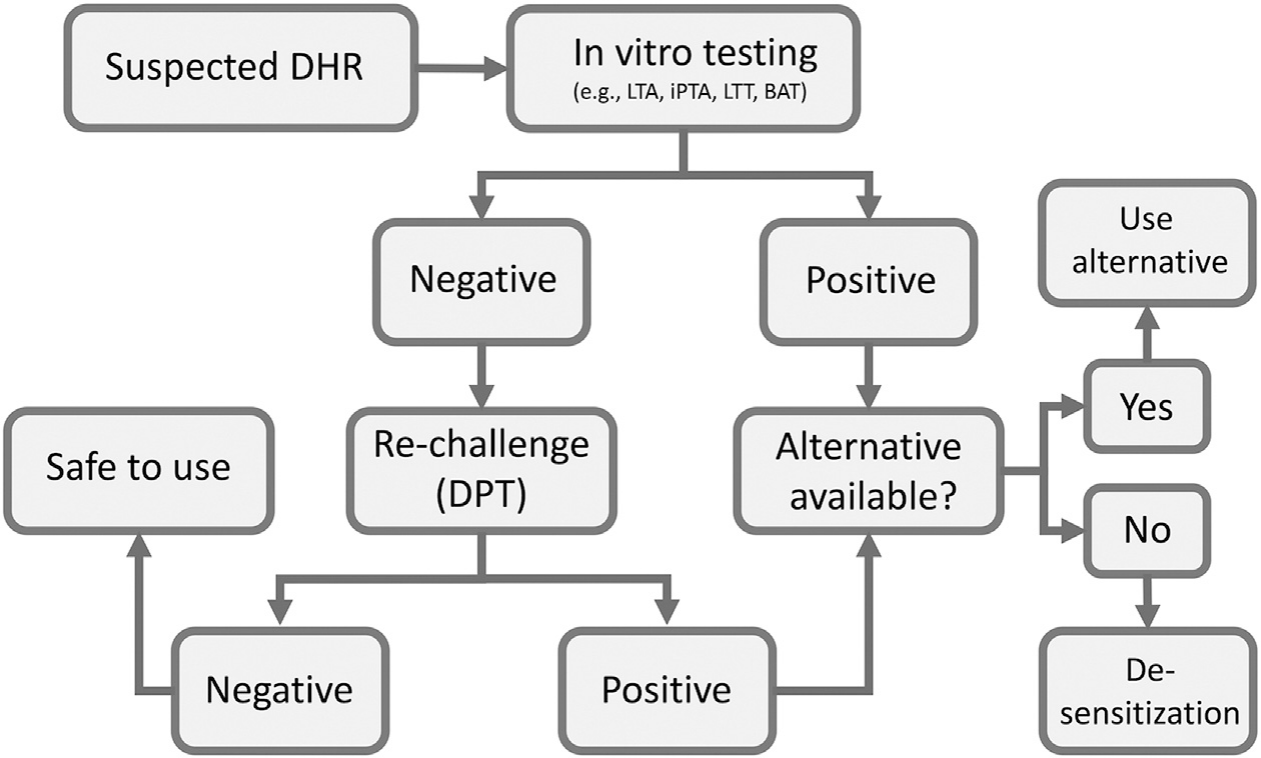
Flow chart of clinical assessment of suspected DHRs. BAT, basophil activation test; DPT, drug provocation test; iPTA, *in vitro* platelet toxicity assay; LTA, lymphocyte toxicity assay; LTT, lymphocyte transformation test.

Drug provocation test (DPT) or drug re-challenge is the controlled administration of the suspected drug under close medical observation for the purpose of diagnosing DHRs. DPT is considered by many guidelines in the field as the “gold standard” for DHRs diagnosis (Aberer et al., 2003). However, the main limitation of the test is possibility of provoking a full reaction, which makes it contraindicated in cases of severe life-threatening DHRs. Therefore, it is only performed if other *in vitro* tests are negative or cannot confirm diagnosis (Figure 2). The test is also contraindicated for pregnant patients and patients with severe comorbidities that put them at high risk. Co-medication with drugs that may interfere with emergency treatments (e.g., adrenergic beta-blockers), mask symptoms of positive response (e.g., H_1_ antihistamines, corticosteroids, ipratropium bromide, leukotriene modifiers and long acting theophylline) or aggravate the reaction (e.g., ACE inhibitors) is contraindicated during DPT (Messaad et al., 2004). Prior to deciding to perform the test a careful medical history of the patient is crucial to determine the nature of previous exposure and reaction type. For patients on multiple drugs, identifying the most likely causative drug can be aided by determining the temporal relationship between the time of the drug administration and the start of the reaction. Also, knowledge of the drugs that are most commonly associated with DHRs and clinical experience with the clinical presentation of the disease is very helpful in zeroing on the culprit drug. Some scoring algorithms are available that may help identifying drugs that are most likely be the cause of the reactions (Naranjo et al., 1981) and a standardized questionnaire has been published (Demoly et al., 1999). The drug should, if possible, be given using the same route of administration that was originally used (i.e., oral, parenteral, topical), and should be started at low dose especially in case of severe reactions and stopped once the first signs of positivity appear (Bousquet et al., 2008).

The positivity of DPT for suspected antimicrobials and NSAIDs-induced DHRs is surprisingly low. In a single center study Vezir *et al.*(Vezir et al., 2014) reported a positivity rate of 6.8% indicating that medical history and clinical presentation are not reliable for diagnosis of DHRs and that DPT can be very useful to rule out suspected drugs.

## *In vitro* Testing Model

*In vitro* testing for DHRs has the advantage of carrying no potential harm to patients (Elzagallaai et al., 2009; Elzagallaai et al., 2011a). The selection of an *in vitro* diagnostic test for DHRs depends on the type of reaction (i.e., immediate vs delayed). Immediate IgE-mediated reactions are mediated by a specific IgE against the culprit drug and, therefore, quantification of those antibodies has been used to diagnose this type of reactions. Radioallergosorbent test (RAST), cellular fluorescent assay-IgE (CAP-IgE) and enzyme-linked immunosorbent assay (ELSA) have a good predictive value (Edwards et al., 1982). A radioactive technique is no longer used but, “RAST” has become generic name for IgE quantification. These tests tend to have an excellent specificity but very poor sensitivity and have been validated only for a few very specific drug-induced reactions (i.e., classical allergy or Gell and Coombs Type I Hypersensitivity) and only for a few drugs (Elzagallaai et al., 2011a). The basophil activation test (BAT) has been found useful in diagnosing immediate-type reactions to muscle relaxants, beta-lactam antibiotics and NSAIDs (Abuaf et al., 1999; Torres et al., 2004; Sanz et al., 2005; De Weck et al., 2009). The lymphocyte transformation test (LTT) measures drug specific T-cells in the circulation and it has been found to useful to aid diagnosis of delayed-type hypersensitivity reactions. However, due to its complected and expensive procedure, its clinical utility has been limited to highly sophisticated research center (Elzagallaai et al., 2011a). The lymphocyte toxicity assay (LTA) and the *in vitro* platelet toxicity assay (iPTA) are both very useful for delayed-type reactions and have been validated for DHRs due to multiple drug classes (Elzagallaai et al., 2010; Elzagallaai et al., 2011b; Elzagallaai et al., 2013). Both tests measures accumulation of toxic reactive drug metabolites (RMs) in peripheral blood monocytes (PBMCs) isolated from the patient and a healthy control. Cells that accumulate higher concentrations of RMs and lack defense against oxidative stress generated by these RMs undergo cell death (necroptosis), which can be measured using different techniques and expressed as percentage of control (vehicle without the drug) (Elzagallaai et al., 2009). A cut-off value of 20% increase in cell death is used to identify positive tests. It is not well characterized how accumulation of RMs lead to eliciting immune-mediated ADR, but it seems to be the first trigger in the cascade of events leading to the reaction manifestations (Cho and Uetrecht, 2017).

The available *in vitro* tests tend to have good specificities and positive predictive values, but their sensitivities and negative predictive values are much affected by the test procedure and the readout systems (Elzagallaai et al., 2009; Elzagallaai et al., 2011a). Other factors that may affect the performance of *in vitro* testing include the time of testing in relation to the beginning of the reaction, the severity of the reaction and type of drug involved (Elzagallaai et al., 2009). Our experience with the recently developed iPTA supports its enhanced sensitivity but more work is needed to define its role in the diagnosis of DHRs (Elzagallaai et al., 2011b; Elzagallaai et al., 2013). These *in vitro* models may serve as logical first step in the management of DHRs (Figure 2). Their main limitation if their technical complexity, availability and cost, which make them confined to well-equipped sophisticated research centers with adequate expertize to perform and interpret the tests. We have been using the *in vitro* toxicity assays (the LTA and later the iPTA) for over 25 years and found them very useful and practical with reasonable turnaround time. In our experience with some care blood samples can be packaged and shipped internationally with good stability and minimal cost. (Table 2)

**TABLE 2:** *In vitro* test used for DHRs and their advantages and limitations.

Test	Advantages	Limitations
Lymphocyte toxicity assay (LTA)	•Can be performed before, during or after the reaction (i.e., used for prediction and diagnosis of DHRs)	•Complicated procedure that is both labor intensive and costly
	•It detects the genetic predisposition of the patient to develop DHRs	•Mainly confined to well-equipped research centers
	•Several drugs can be tested at the same time	•Its predictive value largely depends on the suspected drug
	•Can be used to identify the culprit drug among multiple drugs	•Has been validated for only a small number of drug classes
*In vitro* platelet toxicity assay (*i*PTA)	•Can be performed before, during or after the reaction (i.e., used for prediction and diagnosis of DHRs)	•Recently developed and therefore, lacks inter-lab validation
	•Has simpler and less expensive procedure than the LTA.	
	•Its predictive value seems enhanced compared to the LTA.	
	•It detects the genetic predisposition of the patient to develop DHRs	
	•Can be used to identify the culprit drug among multiple drugs	
Lymphocyte transformation test (LTT)	•Can be used for both immediate-and delayed-type DHRs	•Special technical skills and equipment are required making the test available in only few research centers
		•Low sensitivity
	•Several drugs can be tested at the same time	•It can only be used after the reaction has occurred after recovery, therefore; cannot be used to screen vulnerable patients
	•New readout systems (flow cytometry) have eliminated the need to use radioactive reagents	•Sensitivity and specificity depend on the drug involved and type of reaction
Basophil activation test (BAT)	•Very useful for diagnosis of suspected immediate-type DHRs	•Its procedure has not been standardized and inter-lab variabilities exist
	•Recent flow cytometry applications have enhanced the test sensitivity	•Positive tests decrease over time post-reaction
	•Commercial kits are now available	
	•Has been used successfully for penicillins, NMBAs, NSAIDs, fluoroquinolones and RCMs	

## Animal Models

Use of animal models to evaluate age-specific risk of toxicity is practical and may guide dosing in human children. In addition, the shorter life span of laboratory animals permits detection of long-term effects of toxicities that is normally appears after decades in human subjects. The pitfalls of using animal models to predict drug safety in human children include variability in systems development among animals species and lack of validated animal models for many drug classes (Berde and Cairns, 2000). DHRs are idiosyncratic in nature, which makes them unpredictable. Animal models to predict DHRs would be a very attractive tool for drug developers and health care providers, however, finding a suitable animal model for DHRs has so far proven to be illusive. Animals do develop hypersensitivity reactions to drugs and other xenobiotics but with the same unpredictability as in people (Bloom, 2006; Voie et al., 2012). The pathophysiology underlying DHRs is not fully understood and multiple metabolic and immunologic factors are thought to contribute to the development of these types of reactions.

Attempts at validating animal models to study or predict DHRs have not been very successful (Uetrecht, 2006). One exception to that is the female Brown Norway rats which has been demonstrated to be a model for nevirapine-induced skin rash model (Shenton et al., 2003). In this model Shenton *et al.* (Shenton et al., 2003) were able to induce skin rash in 32/32 (100%) of female Brown Norway rats with nevirapine at dose of 150 mg/kg/day. Interestingly, lower doses of 40–75 mg/kg/day did not lead to skin rash development and protected treated rats from nevirapine-induced skin rash when treated afterward with 150 mg/kg/day (Shenton et al., 2003). Further studies on this model demonstrated that a hydroxy metabolite of nevirapine (12-OH-neverapine) is responsible for the skin rash reaction (Sharma et al., 2013).

The usefulness of an animal model to study a disease depends on how closely the model resembles the actual condition (Scarpelli, 1997). In case of DHRs the clinical presentation of the disease is poorly defined making designing an animal model for the condition an unreachable task without more phenotypic clarity. An additional factor is that patients may develop DHRs to multiple drugs and a reaction to the same drug may present in different ways in different people and, sometimes, in the same patient (Perez-Ezquerra et al., 2006; Pichler et al., 2017). All these factors have hampered any progress in developing animal models to investigate or predict DHRs. This has been a major impediment in research in this area.

## Model-Based Evaluation

Figure 1 summarizes a scheme for evaluation and management of DHRs. Medical history, blood work, allergy work up and scoring algorithms are all utilized early on to assess the probability of a DHR. *In vitro* tests are the first choice giving their safety to the patient. *In vitro* tests often have high specificity and positive result exclude DPT. However, after considering the contraindications, DPT can be performed if the *in vitro* test used is negative. Negative DPT indicates that the drug is safe to use. Positive *in vitro* test or DPT mandate that an alternative drug must be considered. If no alternative drug is available, desensitization procedure should be attempted, a process that depends on the clinician judgment on case-by-case basis.

## Conclusion

In terms of drug development, use of modeling for assessment of drug safety for antimicrobials has been hampered by both a lack of suitable animal models and by a lack of understanding of the putative pathophysiology of DHRs. As our understanding of the fundamental biology of DHRs expands and our ability to develop humanized animal increases it is hoped that this will enable better modeling of DHRs to antimicrobial therapy in children. This review summarizes the current state-of-the-art knowledge of model-based evaluation of DHRs to antimicrobials in children. Several key issues in the field have been highlighted, which include lack of animal model to study the molecular pathophysiology of DHRs and limited validated *in vitro* tests with good predictive values. We believe that further understanding of the exact pathophysiology underlying DHRs will allow the development of more predictive models to optimize the management of these ADRs.
